# Crystal structure of the kringle domain of human receptor tyrosine kinase-like orphan receptor 1 (hROR1)

**DOI:** 10.1107/S2053230X22003855

**Published:** 2022-04-22

**Authors:** Salvatore R. Guarino, Antonella Di Bello, Martina Palamini, Maria Chiara Capillo, Federico Forneris

**Affiliations:** a The Armenise-Harvard Laboratory of Structural Biology, Department of Biology and Biotechnology, University of Pavia, Via Ferrata 9A, 27100 Pavia, Italy

**Keywords:** receptor tyrosine kinases, cancer, kringle domains, human ROR1, immunotherapy

## Abstract

Receptor tyrosine kinase-like orphan receptors (RORs) are monotopic membrane proteins belonging to the receptor tyrosine kinase (RTK) family. RTKs play a role in the control of most basic cellular processes, including cell proliferation, differentiation, migration and metabolism. New emerging roles for RORs in cancer progression have recently been proposed: RORs have been shown to be overexpressed in various malignancies but not in normal tissues, and moreover an abnormal expression level of RORs on the cellular surface is correlated with high levels of cytotoxicity in primary cancer cells. Monoclonal antibodies against the extracellular part of RTKs might be of importance to prevent tumor cell growth: targeting extracellular kringle domain molecules induces the internalization of RORs and decreases cell toxicity. Here, the recombinant production and crystallization of the isolated KRD of ROR1 and its high-resolution X-ray crystal structure in a *P*3_1_21 crystal form at 1.4 Å resolution are reported. The crystal structure is compared with previously solved three-dimensional structures of kringle domains of human ROR1 and ROR2, their complexes with antibody fragments and structures of other kringle domains from homologous proteins.

## Abstract 2

The recombinant production, crystallization and high-resolution X-ray crystal structure of the isolated extracellular kringle domain of human receptor tyrosine kinase-like orphan receptor 1 are reported, together with its comparison with previously solved three-dimensional structures of other kringle domains and their complexes with antibody fragments.

## 1. Introduction

Receptor tyrosine kinase-like orphan receptors (RORs) are poorly characterized receptor tyrosine kinases (RTKs) that are involved in embryonic development and muscle differentiation. The human *ROR1* and *ROR2* genes were originally isolated in a PCR-based screen for RTKs similar to the TRK neurotrophin receptors (Masiakowski & Carroll, 1992 ▸). Simultaneously, PCR screens yielded *ROR*-like genes from *Drosophila melanogaster* (Wilson *et al.*, 1993 ▸), *Mus musculus* (Oishi *et al.*, 1999 ▸; DeChiara *et al.*, 2000 ▸) and *Rattus norvegicus* (Masiakowski & Carroll, 1992 ▸). Despite the limited insight into their biological roles, the highly observed overexpression of *ROR* genes in a range of hematologic and solid malignancies represents a very interesting feature of these macromolecules, which are increasingly becoming an attractive target for immunotherapy (Hojjat-Farsangi, 2014 ▸; Ghaderi *et al.*, 2020 ▸; Borcherding *et al.*, 2014 ▸; Fukuda *et al.*, 2008 ▸).

**Figure 1:**
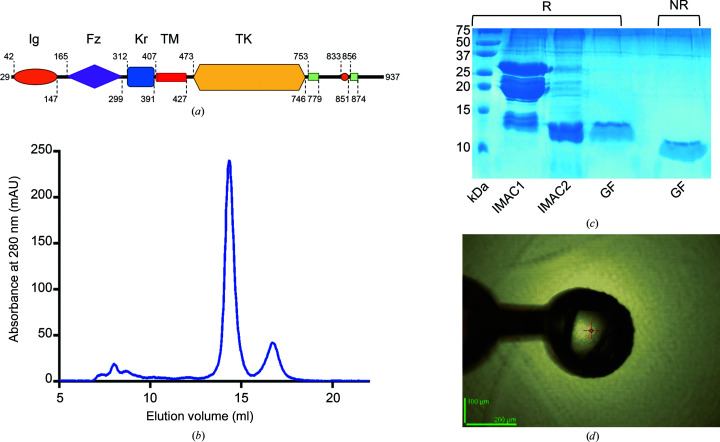
Features, purification and crystallization of hROR1-KRD. (*a*) Domain organization of human ROR1. (*b*) SEC chromatogram of hROR1-KRD. (*c*) SDS–PAGE analysis of hROR1-KRD purification, showing the sample before (IMAC1) and after (IMAC2) proteolytic removal of the 8×His-SUMO tag, plus the final homogeneous hROR1-KRD protein sample after gel filtration (GF) under reducing (R) and nonreducing (NR) conditions. (*d*) The hROR1-KRD crystal used for high-resolution data collection mounted on the MASSIF-3 beamline at ESRF.

At the protein level, the molecular organization of RORs encompasses three main regions: the N-terminal extracellular portion is composed of an immunoglobulin (Ig)-like domain, a Frizzled-like cysteine-rich domain (FZD) and a kringle domain (KRD) (Fig. 1 ▸ *a*). The Ig domain has been proposed to stabilize contacts between RORs and other factors (Saleh *et al.*, 2019 ▸). The FZD is known to bind Wnt5a, with possible involvement in noncanonical Wnt signaling pathways (Konopelski Snavely *et al.*, 2021 ▸; Mikels & Nusse, 2006*a* ▸,*b* ▸; Paganoni *et al.*, 2010 ▸). The cysteine-rich KRD is presently lacking a precise binding partner for hRORs, but homologous domains are frequently key contact platforms in protein–protein and protein–ligand interactions in coagulation proteins, apo­lipoproteins and growth factors (Borcherding *et al.*, 2014 ▸; Stephens *et al.*, 1992 ▸; Mizuno *et al.*, 1994 ▸; Mathews *et al.*, 1996 ▸). A single transmembrane helix connects the extracellular region to the intracellular C-terminus, characterized by a tyrosine kinase domain and additional short segments that are rich in serine/threonine and proline residues (Forrester, 2002 ▸; Borcherding *et al.*, 2014 ▸; Fig. 1 ▸ *a*).

Due to its interaction with Wnt ligands, possible roles have been suggested for ROR1 in Wnt signaling, implicating this molecule in the possible regulation of cellular proliferation, migration and polarization during skeletal, cardiorespiratory and neurological development (Borcherding *et al.*, 2014 ▸; Mikels & Nusse, 2006*b* ▸; Balakrishnan *et al.*, 2017 ▸; Green *et al.*, 2008 ▸). Other reports have also suggested the involvement of ROR1 in skeletal muscle regeneration in adult tissues (Kamizaki *et al.*, 2017 ▸; Karvonen *et al.*, 2018 ▸). However, the most relevant insights regarding the implications of RORs have emerged from the evidence of *ROR* gene overexpression during cancer progression, in particular in the development of solid and hematopoietic malignancies (Hojjat-Farsangi *et al.*, 2014 ▸; Cui *et al.*, 2016 ▸; Daneshmanesh *et al.*, 2013 ▸; Zhang *et al.*, 2012 ▸). Indeed, the strongly restricted expression of *ROR1* in cancer cells and its low level of expression in healthy adult tissues (Balakrishnan *et al.*, 2017 ▸) suggested that ROR1 could be an interesting marker for targeted cancer therapy (Fukuda *et al.*, 2008 ▸; Fauvel & Yasri, 2014 ▸). Parallel to drug-discovery campaigns focusing on the development of small molecules targeting the intracellular kinase domain of ROR2 (Borcherding *et al.*, 2014 ▸; Fauvel & Yasri, 2014 ▸; Rebagay *et al.*, 2012 ▸), the recent characterization of antibodies specifically targeting the KRDs of human ROR1 and ROR2 that display selective antitumor activity *in vitro* and *in vivo* in mice (Qi *et al.*, 2018 ▸; Goydel *et al.*, 2020 ▸) has boosted interest in these orphan receptors, and in particular in their ectodomain architecture. In this context, here we report the recombinant expression, crystallization and structure determination at high resolution of the isolated KRD of human ROR1 (hROR1-KRD).

## 2. Materials and methods

### 2.1. Macromolecule production

#### 2.1.1. Molecular cloning and recombinant expression

The codon-optimized cDNA construct encoding human hROR1-KRD (UniProt entry Q01973, residues 312–393) was synthesized by Genewiz and subcloned into a modified pET-28b-SUMO recombinant expression plasmid (Novagen) using in-frame 5′-BamHI and 3′-NotI restriction sites, yielding a final construct bearing an N-terminal 8×His-SUMO fusion (Table 1 ▸) and cloning scars corresponding to the amino-acid sequences encoded by the restriction nucleases BamHI at the N-terminus (Gly-Ser) and NotI at the C-terminus (Ala-Ala-Ala); the latter precedes an in-frame stop codon already embedded in the expression vector. Subsequently, the plasmid was transformed into *Escherichia coli* T7 Shuffle cells (New England Biolabs) and a single colony was picked and inoculated into 100 ml Luria–Bertani medium supplemented with 0.1 mg ml^−1^ kanamycin [1:1000(*v*:*v*)]. This pre-culture was grown overnight at 30°C in a shaking incubator at 200 rev min^−1^. The following day, this preculture was used to inoculate 6 l autoinducing ZYP5052 medium (Studier, 2005 ▸) for large-scale production. The culture was grown for 4.5 h at 30°C; the culture temperature was then decreased to 20°C and the culture was left for a total of 24 h prior to cell harvesting.

#### 2.1.2. Recombinant protein purification

The bacterial cells were harvested by centrifugation at 4000*g* for 20 min. The cell pellet was resuspended and homogenized in 100 ml buffer *A* (25 m*M* HEPES–NaOH, 0.5 *M* NaCl pH 8) in a 1:5(*w*:*v*) wet cell pellet:buffer ratio and was then disrupted by sonication (16 cycles; 9 s on, 6 s off pulses). The cell debris was removed by high-speed centrifugation (50 000*g*, 40 min, 4°C); the supernatant was then filtered through a 1 µm syringe-driven filter (Minisart GF, Sartorius). The clarified lysate was loaded onto a 5 ml prepacked HisTrap Excel column (GE Healthcare) pre-equilibrated with buffer *A* at a flow rate of 2 ml min^−1^ using an ÄKTApurifier fast protein liquid chromatography (FPLC) system (GE Healthcare). After extensive washing with buffer *A*, elution was carried out stepwise by adding imidazole (25 m*M* to remove nonspecifically bound contaminants, followed by 250 m*M* to elute the protein of interest). Fractions containing His-SUMO-hROR1-KRD, as assessed by SDS–PAGE analysis, were pooled, supplemented with 3 µg ml^−1^ SUMO protease [1:300(*v*:*v*)] and dialyzed overnight at 4°C against buffer *A* to remove excess imidazole and simultaneously cleave the affinity-purification tags. After dialysis, the sample was centrifuged (4500*g*, 4°C, 20 min) and loaded onto a HisTrap Excel column (GE Healthcare) pre-equilibrated with buffer *A* with a 2 ml min^−1^ flow rate. Cleaved ROR1-KRD eluted in the flowthrough fraction. The sample was concentrated to 5.3 mg ml^−1^ using a 3 kDa Amicon Ultra-15 centrifugal filter concentrator (Merck). The concentrated sample was further polished by size-exclusion chromatography (SEC) using a Superdex 75 Increase 10/300 GL column (GE Healthcare) pre-equilibrated with SEC buffer (25 m*M* HEPES–NaOH, 200 m*M* NaCl pH 8). hROR1-KRD peak fractions underwent SDS–PAGE analysis to assess the final sample purity; they were further concentrated to 13.6 mg ml^−1^ and used immediately for crystallization.

### 2.2. Crystallization

Purified hROR1-KRD at 13.6 mg ml^−1^ was subjected to extensive crystallization screening against a broad series of commercially available crystallization screens (Molecular Dimensions) using the sitting-drop vapor-diffusion method. Nanolitre-scale droplets (150 nl protein + 150 nl reservoir) were set up using an Oryx 8 robotic nanodispenser (Douglas Instruments) in MRC 96-well PS plates (SWISSCI). Initial hits found in conditions from the JCSG+ screen underwent manual optimization using the sitting-drop method by dispensing droplets comprised of 1 µl protein solution and 1 µl reservoir solution into MRC Maxi 48 PS plates (SWISSCI). The best diffracting crystals of hROR1-KRD were obtained after 25 days at 20°C in conditions consisting of 0.8–1.2 *M* sodium citrate tribasic dihydrate, 0.1 *M* sodium cacodylate pH 6.5 (Table 2 ▸). Crystals were harvested using plastic litholoops (MiTeGen), cryoprotected using 20%(*v*/*v*) glycerol and flash-cooled in liquid nitrogen prior to data collection.

### 2.3. Data collection and processing

X-ray diffraction data were collected from a single crystal at 100 K on the MASSIF-3 (ID30A-3) beamline at the ESRF synchrotron in Grenoble, France. The data set was recorded on an EIGER 4M detector (Dectris) at a wavelength of 0.9677 Å (12.812 keV) and a crystal-to-detector distance of 99.86 mm. A total of 1800 images were collected with an exposure time of 0.002 s, a rotation range of 0.1° and 10% beam transmission. Data were indexed and integrated with *XDS* (Kabsch, 2010 ▸), followed by scaling and merging using *AIMLESS* (Evans & Murshudov, 2013 ▸). Data-collection statistics are given in Table 3 ▸.

### 2.4. Structure solution and refinement

The crystal structure of hROR1-KRD was solved by molecular replacement using a search model generated by combining the structures of hROR1-KRD extracted from the complex with an antibody fragment (PDB entry 6ba5; Qi *et al.*, 2018 ▸) and of free KRD from human ROR2 (PDB entry 6osn; Goydel *et al.*, 2020 ▸) as a search model. The resulting model was used in molecular replacement with *Phaser* (McCoy *et al.*, 2007 ▸), yielding a solution comprising a single copy of hROR1-KRD in the asymmetric unit. The structure was refined by alternating steps of manual building in *Coot* (Emsley *et al.*, 2010 ▸) and automated refinement with *phenix.refine* (Adams *et al.*, 2010 ▸) and *REFMAC* 5.8.0267 (Murshudov *et al.*, 2011 ▸). Because of the high resolution of the data, an anisotropic *B*-factor model was used in combination with TLS refinement. Model validation and Ramachandran analysis were performed using *MolProbity* (Chen *et al.*, 2010 ▸) and the validation tools available on the Protein Data Bank server (Gore *et al.*, 2017 ▸). Final refinement statistics are summarized in Table 4 ▸. Structural figures were prepared with *PyMOL* (http://www.pymol.org). Superpositions were performed using the ‘super’ command in *PyMOL*. All-atom r.m.s.d. values were computed accordingly.

## 3. Results and discussion

### 3.1. Production of recombinant hROR1-KRD

As RORs are increasingly becoming interesting targets for cancer immunotherapy (Fauvel & Yasri, 2014 ▸; Menck *et al.*, 2021 ▸; Wu *et al.*, 2019 ▸), we aimed to produce the isolated hROR1-KRD antigen and characterize it by solving its crystal structure. Using the reference sequence of human ROR1 found in UniProt, we designed a sequence-optimized DNA construct for hROR1-KRD encompassing residues 312–393 and subcloned it into a series of *E. coli* recombinant expression vectors bearing various affinity and solubility tags. The use of *E. coli* T7 Shuffle K12 cells was key to successful protein production, as this bacterial strain is engineered to facilitate the correct folding and stabilization of recombinant proteins bearing multiple disulfide bonds. After several attempts, we successfully produced hROR1-KRD in a soluble form using an expression vector bearing an N-terminal 8×His-SUMO tag. SDS–PAGE analysis after the first immobilized Ni^2+^ affinity-chromatography step revealed multiple bands corresponding to the desired 8×His-SUMO-hROR1-KRD construct (23 kDa), the SUMO tag (13 kDa) and cleaved ROR1-KRD (10 kDa), suggesting partial spontaneous tag proteolysis during the purification steps (Supplementary Fig. S1*a* ). A further SUMO protease digestion of the eluted fraction completed tag cleavage. The digested fractions were then purified by reverse immobilized Ni^2+^-affinity chromatography to collect hROR1-KRD in the flowthrough fraction (Supplementary Fig. S1*b* ) A final purification step by size-exclusion chromatography allowed the removal of large oligomers (Fig. 1 ▸ *b*) and highlighted the monomeric state of the protein in solution. SDS–PAGE analysis confirmed the correct formation of disulfide bonds for the main protein fraction, as shown by the extended band migration observed under nonreducing conditions compared with their reducing counterparts (Fig. 1 ▸ *c*). The fractions corresponding to pure homogeneous hROR1-KRD were used in crystallization experiments.

### 3.2. Crystal structure of hROR1-KRD

**Figure 2:**
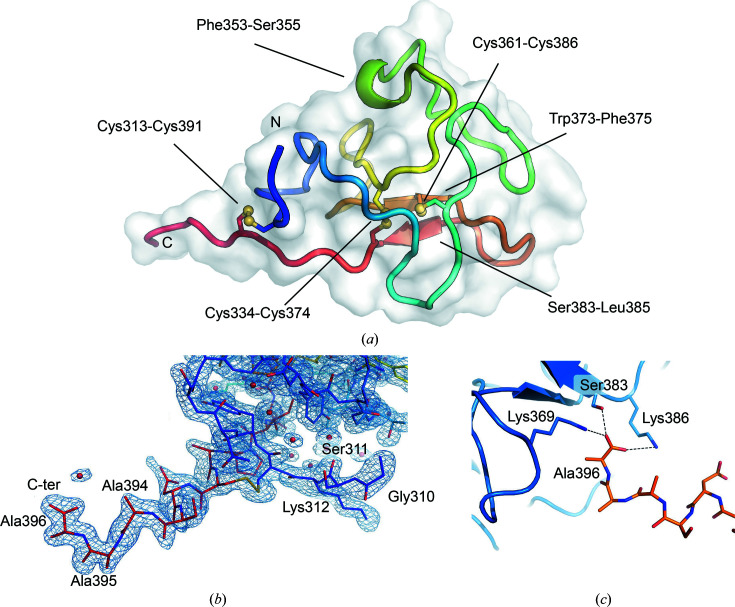
Details of the three-dimensional structure of hROR1-KRD. (*a*) Cartoon representation of hROR1-KRD. The polypeptide chain is colored from the N-­terminus (blue) to the C-terminus (red). Disulfide bonds are shown in ball-and-stick representation and residues constituting the boundaries of the secondary-structure elements are labeled. (*b*) Electron density (blue, 2*F*_o_ − *F*_c_, contour level 1.0σ) of the regions describing the amino-acid residues of the restriction cloning scars at the N-termini (blue) and C-termini (red) of hROR1-KRD. First-shell water molecules are shown as red spheres. (*c*) Details of the crystal contact interactions of the C-terminal residue Ala396 (orange) with side chains of a neighboring molecule in the crystal packing (blue).

After extensive crystallization screening, we obtained multiple hits in the JCSG+ screen (Molecular Dimensions). Optimization led to large three-dimensional crystals (with average dimensions of 50 × 100 × 200 µm; Fig. 1 ▸ *d*). A single hROR1-KRD crystal allowed complete X-ray data collection and structure determination at very high resolution (1.4 Å). The crystal belonged to the trigonal space group *P*3_2_21, with unit-cell parameters as indicated in Table 3 ▸. The Matthews coefficient (*V*_M_) of 2.47 Å^3^ Da^−1^ assumes the presence of 50% solvent and one hROR1-KRD molecule in the asymmetric unit. The phase problem could be solved by molecular replacement using an ensemble model generated by superimposing the model of the ROR1 kringle domain extrapolated from its complex with an antitumor bispecific antibody fragment (PDB entry 6ba5; Qi *et al.*, 2018 ▸) and the isolated ROR2 kringle domain (PDB entry 6osn; Goydel *et al.*, 2020 ▸). The molecular structure could be modeled in the electron density from the N-terminus to the C-terminus without interruptions (including restriction cloning scars) and was refined to final *R*_work_ and *R*_free_ values of 0.221 and 0.214, respectively. The complete refinement statistics are reported in Table 4 ▸. The hROR1-KRD structural organization presents the typical features of kringle domains, characterized by a globular architecture of overall triangular shape with very poor secondary structure except for a short antiparallel β-sheet comprising residues Trp373–Phe375 and Ser383–Leu385 and a small α-helical turn defined by residues Pro353–Leu355 (Fig. 2 ▸ *a*). The three conserved KRD disulfide bonds are found in the structure, involving Cys313–Cys391, Cys334–Cys374 and Cys361–Cys386 (Fig. 2 ▸ *a*). The amino-acid residues due to restriction cloning scars Gly310-Ser311 at the N-terminus and Ala394-Ala395-Ala396 at the C-terminus are covered by electron density (Fig. 2 ▸ *b*) and point outside the globular region of hROR1-KRD. The extended C-terminal sequence is involved in crystal contacts with a neighboring molecule, with the carboxy-terminus of Ala396 forming hydrogen-bonding interactions with the side chains of Lys369, Lys382 and Ser383 (Fig. 2 ▸ *c*).

### 3.3. Comparison with related kringle domains

**Figure 3:**
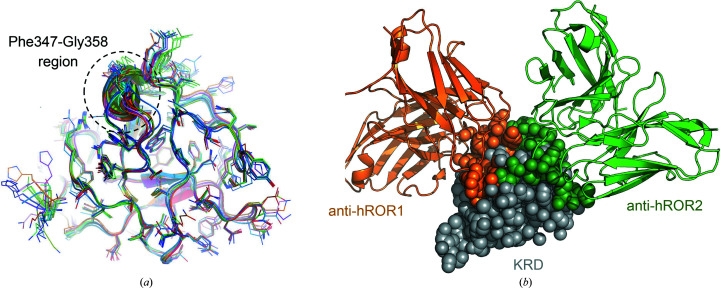
Structural comparison of hROR1-KRD with KRDs from related ROR1/2 structures. (*a*) Comparison of hROR1-KRD with related kringle domains: the most relevant deviations were found in the two consecutive turns encompassing the Phe347–Gly358 region (black dashed circle). ROR1 structures from PDB entries 5z55 (marine), 6ba5 (dark blue, cyan, sky blue and purple-blue for the four chains in the asymmetric unit) and 6ban (green forest, split pea and lime green for the four chains in the asymmetric unit) and ROR2 structures from PDB entries 6osh (orange), 6osn (magenta) and 6osv (pink) are shown. (*b*) Superposition of the structures of ROR1 and ROR2 KRDs in complex with therapeutic antibody fragments (orange for ROR1 and green for ROR2) reveals the presence of two juxtaposed epitopes located on the domain surface.

**Figure 4:**
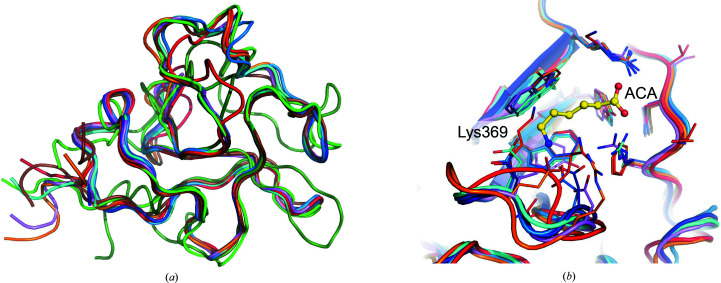
Comparison of hROR1-KRD with structurally related KRDs. (*a*) Cartoon representation of the superposition of hROR1-KRD with the closest structurally related KRDs from homologous proteins identified by *DALI* and *PDBeFold*, as detailed in Supplementary Table S1: PDB entries 5ct3 (cyan), 1i71 (dark blue), 1krn (pink), 2doh (light green), 6rx7 (orange), 1nl2 (brown), 1kiv (light blue), 6oqk (dark green) and 2k51 (red). (*b*) Details of the nonconserved lysine residue at position 369 in hROR1-KRD (shown in red), occupying a site displaying a negatively charged residue used by HGF/SF (orange), plasminogen (dark blue) and apolipoprotein KRDs (cyan, pink and light blue) to bind lysine and its analogs [aminocaproic acid (ACA) from PDB entry 2pk4 is shown in yellow ball-and-stick representation].

We compared our structure of the isolated hROR1-KRD with those found in the structures of the complexes with antibody fragments (PDB entries 6ba5 and 6ban; Qi *et al.*, 2018 ▸), with the structure of the same domain obtained using NMR (PDB entry 5z55; Ma *et al.*, 2018 ▸) and with the structures of the KRD of the closest paralog ROR2 (58% sequence identity overall, 62% for the KRDs) in its free state (PDB entry 6osn; Goydel *et al.*, 2020 ▸) and in complex with antibody fragments (PDB entries 6osh and 6osv; Goydel *et al.*, 2020 ▸). As expected, the superpositions showed nearly identical arrangements, with root-mean-square deviations (r.m.s.d.s) on all atoms ranging from 0.28 to to 0.55 Å. The most relevant deviations were found in the two consecutive turns encompassing residues Phe347–Gly358 (Fig. 3 ▸ *a*); the maximum displacement observed in this region (for C^α^ atoms) was 3.3 Å. These two turns do not constitute the epitope for anti-ROR1 antibodies (Ma *et al.*, 2018 ▸); however, they are part of the site recognized by the recently proposed anti-ROR2 therapeutic antibodies (Goydel *et al.*, 2020 ▸). Interestingly, the two distinct antibody-binding sites are located on two juxtaposed regions of the KRD (Fig. 3 ▸ *b*), further supporting the high potential of this domain as an antigen for immunotherapy. Structure-similarity searches using the *DALI* (Holm *et al.*, 2008 ▸) and *PDBeFold* (Krissinel & Henrick, 2004 ▸) servers enabled additional structural comparisons to identify related KRDs (Fig. 4 ▸ *a*). In particular, hROR1-KRD showed strong similarity to the K1 domain of hepatocyte growth factor (HGF/SF; Sigurdardottir *et al.*, 2015 ▸), KIV domains 2, 7 and 10 of human apolipoprotein A (Ye *et al.*, 2001 ▸; Santonastaso *et al.*, 2020 ▸; Mochalkin *et al.*, 1999 ▸), KRD2 and KRD4 of human plasminogen (PDB entries 6oqk and 1krn; Yuan *et al.*, 2019 ▸; Stec *et al.*, 1997 ▸), KRD1 of prothrombin (Huang *et al.*, 2003 ▸) and the KRD of neurotrypsin (PDB entry 2k51; Ozhogina *et al.*, 2008 ▸) (Supplementary Table S1).

The analysis also highlighted the unusual presence of a nonconserved lysine residue (Lys369) in hROR1-KRD in a region proximate to a hydrophobic pocket generated by the side chains of conserved residues within the two antiparallel β-strands of the KRD protein fold. In the homologous plasminogen, HGF/SF and apolipoprotein A, this position is occupied by negatively charged residues (Asp or Glu), creating a strong binding site for lysine and its analogs (Hochschwender & Laursen, 1981 ▸; Váli & Patthy, 1980 ▸; Wu *et al.*, 1991 ▸) that has been widely explored for its druggability (Sandmark *et al.*, 2020 ▸; Sigurdardottir *et al.*, 2015 ▸; Fig. 4 ▸ *b*). The presence of a lysine residue within this site clearly prevents the ability of ROR1 KRD to bind additional lysine residues, constituting a distinguishing characteristic of this protein domain.

## 4. Conclusions

This work provides a protocol to efficiently produce and characterize hROR1-KRD and highlights the remarkable similarity between paralogous ROR1 and ROR2 KRD structures in free and antibody-bound states, as well as to KRDs found in homologous proteins. The presence of two juxtaposed epitopes located on the KRD surface further emphasizes the versatility and relevance of this extracellular domain for antibody targeting, offering additional opportunities for the development of innovative cancer therapeutics focusing on these orphan receptors.

## Supplementary Material

PDB reference: kringle domain of human receptor tyrosine kinase-like orphan receptor 1, 7tng

Supplenentary Figure and Table. DOI: 10.1107/S2053230X22003855/ow5031sup1.pdf

## Figures and Tables

**Table 1:** Macromolecule-production information

Source organism	*H. sapiens*
DNA source	Synthetic gene, codon-optimized based on UniProt entry Q01973, residues 312–393
Expression vector	Modified pET-28b-SUMO (Novagen)
Expression host	*E. coli* T7 Shuffle K12 cells (New England Biolabs)
Complete amino-acid sequence of the construct produced	MGSSHHHHHHHHSSDSEVNQEAKPEVKPEVKPETHINLKVSDGSSEIFFKIKKTTPLRRLMEAFAKRQGKEMDSLRFLYDGIRIQADQTPEDLDMEDNDIIEAHREQIGGGSKCYNSTGVDYRGTVSVTKSGRQCQPWNSQYPHTHTFTALRFPELNGGHSYCRNPGNQKEAPWCFTLDENFKSDLCDIPACDSAAA
Complete amino-acid sequence of the construct used for crystallization after SUMO protease cleavage	GSKCYNSTGVDYRGTVSVTKSGRQCQPWNSQYPHTHTFTALRFPELNGGHSYCRNPGNQKEAPWCFTLDENFKSDLCDIPACDSAAA

**Table 2:** Crystallization

Method	Sitting-drop vapor diffusion
Plate type	MRC Maxi 48-well PS (SWISSCI)
Temperature (K)	293
Protein concentration (mg ml^−1^)	13.6
Buffer composition of protein solution	25 m*M* HEPES–NaOH, 200 m*M* NaCl pH 8
Composition of reservoir solution	0.8–1.2 *M* trisodium citrate, 0.1 *M* sodium cacodylate pH 6.5
Volume and ratio of drop	1.0 µl, 1:1
Volume of reservoir (µl)	100

**Table 3:** Data collection and processing Values in parentheses are for the outer shell.

Diffraction source	MASSIF-3 [ID30A-3], ESRF
Wavelength (Å)	0.9677
Temperature (K)	100
Detector	EIGER 4M
Crystal-to-detector distance (mm)	99.86
Rotation range per image (°)	0.1
Total rotation range (°)	180
Exposure time per image (s)	0.002
Space group	*P*3_2_21
*a*, *b*, *c* (Å)	48.16, 48.16, 69.02
α, β, γ (°)	90, 90, 120
Mosaicity (°)	0.1
Resolution range (Å)	41.71–1.40 (1.42–1.40)
Total no. of reflections	139688 (5207)
No. of unique reflections	18755 (938)
Completeness (%)	99.8 (99.7)
Multiplicity	7.4 (5.6)
〈*I*/σ(*I*)〉	18.3 (1.7)
CC_1/2_	0.985 (0.737)
*R*_p.i.m._ †	0.063 (0.494)
Overall *B* factor from Wilson plot (Å^2^)	14.3

† *R*_p.i.m._ = 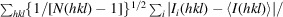 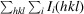 , where *I*_*i*_(*hkl*) is the *i*th observed intensity for a reflection and 〈*I*(*hkl*)〉 is the average intensity obtained from multiple observations of symmetry-related reflections.

**Table 4:** Structure refinement Values in parentheses are for the outer shell.

Resolution range (Å)	41.71–1.40 (1.436–1.400)
Completeness (%)	99.7
No. of reflections, working set	18716 (1291)
No. of reflections, test set	1006 (66)
Final *R*_cryst_	0.211 (0.556)
Final *R*_free_	0.214 (0.615)
No. of non-H atoms	
Total	776
Protein	713
Ligand	0
Solvent	63
R.m.s. deviations	
Bond lengths (Å)	0.018
Angles (°)	2.07
Average *B* factors (Å^2^)	
Protein	19.01
Water	28.43
Ramachandran plot	
Favored regions (%)	98.8
Additionally allowed (%)	1.2
