# Supplementary material for: Crystal structure of the kringle domain of human receptor tyrosine kinase-like orphan receptor 1 (hROR1)
Source: Acta Crystallogr F Struct Biol Commun. 2022 Apr 22;78(Pt 5):185–92. doi: 10.1107/S2053230X22003855 (PMC9067376; doi:10.1107/S2053230X22003855)
Supplement: Supplementary file 1 [file f-78-00185-sup1.pdf]

STRUCTURAL BIOLOGY COMMUNICATIONS

Volume 78 (2022)

Supporting information for article:

Crystal structure of the kringle domain of human receptor tyrosine kinase-like orphan receptor 1 (hROR1)

Salvatore R. Guarino, Antonella Di Bello, Martina Palamini, Maria Chiara Capillo and Federico Forneris

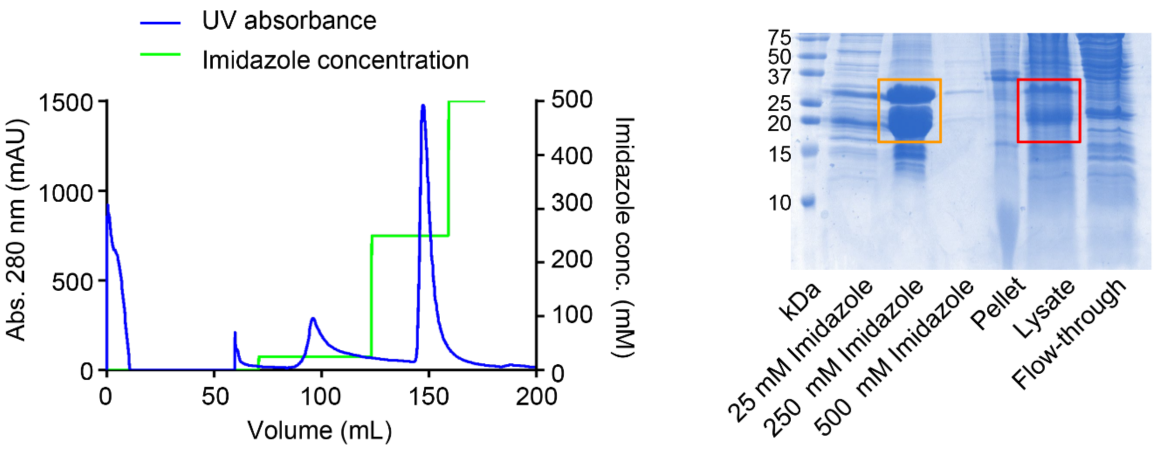

(a)

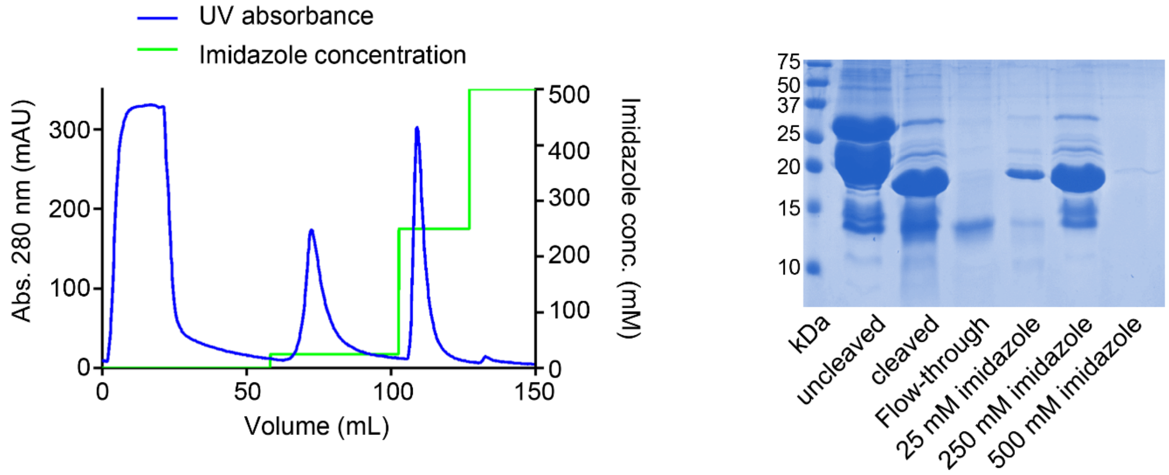

(b)

Supplementary Figure S1 – Details of hROR1-KRD purification.

(*a*) Chromatogram of the first hROR1-KRD purification step, immobilized Ni^{2+} affinity chromatography (left) and associated SDS-PAGE analysis (right): The protein was manly eluted at 250 mM imidazole (50 %B). The SDS-PAGE also confirms the partial spontaneous 8xHis-SUMO tag proteolysis (orange square), observed already in the samples immediately after cell lysis (red square). (b) Chromatogram o the second hROR1-KRD purification step, reverse immobilized Ni^{2+} affinity chromatography (left) and associated SDS-PAGE analysis (right). After removal of the 8xHis-SUMO tag, the protein of interest elutes in flow through fraction. The SDS-PAGE also shows control lanes for the sample before (uncleaved) and after (cleaved) cleavage of the 8xHis-SUMO tag.

Supplementary Table S1

Structurally-related KRDs from homologous proteins identified by DALI and PDBeFold

MOLECULE	PDB ID	RMSD (Å)	SCORE
HGF/SF	5CT3	1.5	14.9 (DALI Z-score)
Apolipoprotein A KIV-7	1I71	1.6	14.7 (DALI Z-score)
Plasminogen KRD4	1KRN	1.2	14.6 (DALI Z-score)
Angiostatin	2DOH	1.6	14.5 (DALI Z-score)
Apolipoprotein A KIV-2	6RX7	1.2	14.4 (DALI Z-score)
Prothrombin	1NL2	1.8	13.8 (DALI Z-score)
Apolipoprotein A KIV-10	1KIV	1.3	13.6 (DALI Z-score)
Plasminogen KRD2	6OQK	1.7	0.20 (PDBeFold Q-score)
Neurotrypsin	2K51	2.1	0.12 (PDBeFold Q-score)
